# Gender difference in arsenic biotransformation is an important metabolic basis for arsenic toxicity

**DOI:** 10.1186/s40360-022-00554-w

**Published:** 2022-02-28

**Authors:** Maihaba Muhetaer, Mei Yang, Rongxiang Xia, Yuanyan Lai, Jun Wu

**Affiliations:** 1 Department of Occupational Health and Environmental Health, Public Health College of Xinjiang Medical University, No.567, Sunde North Road, Shuimogou District, Xinjiang 830011 Urumqi, People’s Republic of China; 2 Department of Biochemistry and Molecular Biology, School of Basic Medical Sciences, Xinjiang Medical University, 830011 Urumqi, People’s Republic of China; 3 Department of Endemic Disease Control, Center for Disease Control and Prevention of Xinjiang Uygur Autonomous Region, 830011 Urumqi, People’s Republic of China

**Keywords:** Arsenic, Gender difference, MMA, DMA, SAM, ARR, NAD, PNP, PK, MPO, As3MT

## Abstract

**Background:**

Arsenic metabolism enzymes can affect the toxic effects of arsenic. However, the effects of different genders on the metabolites and metabolic enzymes in liver arsenic metabolism is still unclear. This study analyzed the gender differences of various arsenic metabolites and metabolic enzymes and further explored the effects of gender differences on arsenic metabolism in liver tissues of rats.

**Methods:**

Rats were treated with high/medium/low doses of iAs^3+^ or iAs^5+^. Liver pathological changes were observed with electron microscopy. The monomethyl aracid (MMA) and dimethyl aracid (DMA) was determined by high performance liquid chromatography-hydride generation atomic fluorescence spectroscopy. S-adenosylmethionine (SAM), arsenate respiratory reductase (ARR), nicotinamide adenine dinucleotide (NAD), purine nucleoside phosphorylase (PNP), pyruvate kinase (PK), and myeloperoxidase (MPO) SAM, ARR, NAD, PNP, PK, and MPO were determined by enzyme-linked immunoassay. RT-qPCR was used to determine Arsenic (+ 3 oxidation state) methyltransferase (AS3MT).

**Results:**

The iAs^3+^ and iAs^5+^ at high doses induced pathological changes in the liver, such as increased heterochromatin and lipid droplets. Compared within the same group, MMA and DMA were statistically significant in iAs^3 +^ high, iAs^3 +^ medium and iAs^5+^ low dose groups (*P* < 0.05). MMA of male rats in iAs^3+^ high and medium groups was higher than that of female rats, and the DMA of male rats was lower than that of female rats. *As3MT* mRNA in the male iAs^3+^ high group was higher than that of females. Besides, compared between male and female, only in iAS^3+^ low dose, iAS^3+^ medium dose, iAS^5+^ low dose, and iAS^5+^ medium dose groups, there was significant difference in SAM level (*P* < 0.05). Compared within the same group, male rats had significantly higher PNP and ARR activities while lower PK activity than female rats (*P* < 0.05). Between the male and female groups, only the iAS^3+^ high dose and medium dose group had a statistically significant difference (*P* < 0.05). The NAD activity of females in iAS^3+^ high dose group was higher than that of males.

**Conclusion:**

The gender differences in the arsenic metabolism enzymes may affect the biotransformation of arsenic, which may be one of the important mechanisms of arsenic toxicity of different sexes and different target organs.

## Background

Arsenic exposure is common, which can be from contaminated drinking water and industrial activities [1]. The toxicity of different forms of arsenic is not only related to the environment, but also closely related to the metabolism and detoxification mechanism of the organism [2]. When inorganic arsenic enters the organism, it is converted into an organic form mainly through methylation in the liver, which is then excreted from the body. Generally, inorganic arsenic enters the body in the form of arsenite (iAs^3+^) or arsenate (iAs^5+^). The iAs^5+^ can be reduced to iAs^3+^, and then undergo methylation and other reduction reactions to form monomethyl aracid (MMA) and dimethyl aracid (DMA). Arsenic (+ 3 oxidation state) methyltransferase (AS3MT) uses S-adenosylmethionine (SAM) as a methyl donor to catalyze the methylation of arsenic [3, 4]. Study has shown that As3mt gene knockout mice changed the main metabolic pathways in a sex-specific manner [5]. Arsenate respiratory reductase (ARR) catalyzes the reduction of arsenate to arsenite [6]. Microbial arsenate respiration enhances the mobility of arsenic, causing poisoning to tens of millions of people worldwide. ARR has been detected in arsenic-contaminated soil and organisms expressing ARR can promote the reduction of dissolved arsenate [7]. The absorption and metabolism of arsenic depends on the polymorphism of the gene encoding purine nucleoside phosphorylase (PNP) [8]. In addition, nicotinamide adenine dinucleotide (NAD), as one of the coenzymes of glycolysis metabolism, greatly enhances the reduction of As^5+^ [9]. Arsenic exposure increases the level of pyruvate kinase M2(PKM2) from week 2 of exposure [10] and promotes a significant increase in serum myeloperoxidase (MPO) activity [11]. The severity of arsenic poisoning is closely related to changes in metabolic enzyme activities. Therefore, exploring changes in metabolic enzyme activity during arsenic poisoning may help to identify individuals who are particularly vulnerable to arsenic toxicity.

The methylation ability of arsenic varies among species, individuals and populations. Previous study has shown that there were significant gender differences in arsenic metabolism [12]. Arsenic exposure is related to gender differences in gene epigenetic regulation [13]. There is literature showing that arsenic methylation was more effective in women than men [14]. In order to explore the difference in methylation ability between male and female rats, in this study, we measured arsenic metabolites and enzyme activity in the liver. Meanwhile, we studied the effects of arsenic, gender, and exposure level on arsenic metabolism in rats.

## Materials and methods

### Animals

Wistar rats (body weight: 80-120 g; age: 1 month old; *n* = 70, including 35 males and 35 females) were from the Experimental Animal Center of Xinjiang Medical University. The animal usage license number was SYXK (new) 2003–0001. All rats were kept in an environment with a relative humidity of 40% to 60% and room temperature of 18 °C to 22 °C. A double-blinded method was used during the allocation, the conduct of the experiment, the outcome assessment, and the data analysis. No rat died during the study. All animal experiments were conducted according to the ethical guidelines of Experimental Animal Center of Xinjiang Medical University. This study was approved by the Ethics Committee of Xinjiang Medical University. All efforts were made to minimize animal suffering. This study is reported in accordance with the ARRIVE (Animal Research: Reporting In Vivo Experiments) guidelines.

### Arsenic poisoning model establishment and animal grouping

After 1 week of adaptive feeding, the rats were randomly divided into 7 groups, namely the normal control (deionized water) group, the low-dose (1/45LD_50_, 2.33 mg/kg)/medium-dose (1/15LD_50_, 6.67 mg/kg)/high-dose (1/5LD_50_), 20.00 mg/kg) iAs^5+^ exposure groups, and the low dose (1/45LD_50_, 2.33 mg/kg)/medium dose (1/15LD_50_, 6.67 mg/kg)/high dose (1/5LD_50_, 20.00 mg/kg) iAs^3+^ groups, with 10 animals in each group, half male and female. NaAsO_2_ (analytical grade; Beijing Third Chemical Reagent Factory, China) was dissolved in distilled water to prepare iAs^3+^ stock solution with the concentration of 0.42 mg/ml. Na_2_AsO_4_ (analytical grade; Hunan Phoenix Chemical Reagent Factory, China) was also dissolved in distilled water to prepare iAs^5+^ stock solution with the concentration of 0.42 mg/ml. Free drinking water was used for the poisoning, and the poisoning was continued for 90 days. The stock solution of iAs^3+^ and iAs^5+^ was re-prepared every two days during the poisoning period. In order to control the amount of water consumed daily, there were two animals (same gender) per cage. During the poisoning period, the animal’s water consumption was recorded daily, and the animal’s body weight was measured every 6 days. The Horn method was used to determine that the oral intake of iAs^3+^ and sodium iAs^5+^ was taken orally in accordance with the mass ratio of sodium arsenite to 1:1 in Wistar rats. According to the average animal body weight, the daily water intake, and the LD_50_ of sodium arsenite metabolism in Wistar rats, the daily amount of iAs^3+^ and iAs^5+^ added to the drinking water of each group of animals was determined. The iAs^3+^ and iAs^5+^ stock solution was diluted according to the average weight of the rats and the daily water intake to ensure the consistency of the arsenic dose.

### Sample collection

After 90 days of exposure, the animals were sacrificed by cervical dislocation. Then the liver was dissected immediately, and rinsed with normal saline at 4 °C. After that, the wet weight of the liver was weighed. Then, some liver tissues (0.3 g) were then homogenized on the ice with an electric homogenizer in PBS (3 mL; PH 7.4). The homogenate was centrifuged at 3000 r/min at 4 °C for 20 min. Then, the supernatant was collected and stored at -80 °C until use. Some liver tissues was fixed with glutaraldehyde and subjected to pathological analysis with the electron microscope, which was performed by the Department of Electron Microscope, School of Basic Medical Sciences, Xinjiang Medical University.

### High performance liquid chromatography-hydride generation atomic fluorescence spectroscopy

The rapid solvent extraction (ASE) method was used for sample pretreatment [15]. The contents of arsenic speciation products (iAs^3+^, iAs^5+^, MMA, DMA) in liver tissues were determined by high performance liquid chromatography-hydride generation atomic fluorescence spectroscopy. The detection limit of the method was 6.67–12.03 μg/L, RSD < 3%. The average recovery rate of DMA in each iAs^3+^group was between 98.9% and 102.9%.

### ELISA

The activities of PK, ARR, MPO, SAM, NAD and PNP were measured with Enzyme-linked immunoassay kits (Shanghai Huole Biological Technology Co, Ltd, Shanghai, China) according to the instructions. PK was expressed as mU/L. The results of ARR, MPO, NAD, and PNP were shown as U/L. The result of SAM was shown as nmol/L.

### Real-time fluorescent quantitative PCR (RT-qPCR)

Total RNA was isolated from liver tissues using the Trizol reagent (Invitrogen, USA). The cDNA was synthesized with a high-capacity cDNA reverse transcription Kit (Fermantas) from 1 μg total RNA. NANODROP 1000 (Thermo) was used to detect the quality and concentration of the extracted total RNA and synthesized cDNA. The PCR procedure for *AS3MT* and *β-actin* was: 95 °C, 2 min; 95 °C, 5 s, and 58 °C, 30 s, 40 cycles. The primers of *AS3MT* were designed and synthesized by TaKaRa (Tokyo, Japan) while those of *β-actin* were designed and synthesized by Sangon Biotech (Shanghai, China). The primer sequences were as follows: rat *AS3MT*: 5’-GGG ACA CAT CAC CGG GAT AGA C-3’ (Forward) and 5’-AAC ATC TCA ATT TGG CCG TGA AG-3’ (Reverse); and rat *β-actin*: 5’-TCC TGT GGC ATC CAT GAA ACT-3’ (Forward) and 5’-GAA GCA TTT GCG GTG CAC GTA-3’ (Reverse). The RT-qPCR was performed on the iQ5 Real-time PCR system (BioRad, Hercules, CA, USA). The reaction system (20 μL in total) included SYBR Green I (TaKaRa Biotechnology (Dalian) Co., Ltd., Dalian, China) 10 μL, upstream primer 0.5 μL, downstream primer 0.5 μL, cDNA 2 μL, and ultrapure water 7 μL. Each sample was analyzed in duplicate and expression of the *AS3MT* mRNA was normalized to that of β-actin. The relative expression of *As3MT* mRNA was calculated by the 2^−△△Ct^ method.

### Statistical analysis

SPSS 17.0 software was used for data analysis. All experiments were repeated three times, and the results were expressed as mean ± standard deviation. The normality of data distribution was analyzed with the Kolmogorov–Smirnov and Shapiro–Wilk test results. For data of normal distribution, one-way variance analysis was used for multi-comparison followed by LSD-t test or SNK method for pairwise comparison. Dunnett's T3  was used when variances are heterogeneous. Kruskal-Wallis H test was used when the data was of non-normal distribution. Pearson correlation method was used for correlation analysis. The *P* < 0.05 was considered as statistically significant.

## Results

### Pathological results

**Fig. 1:**
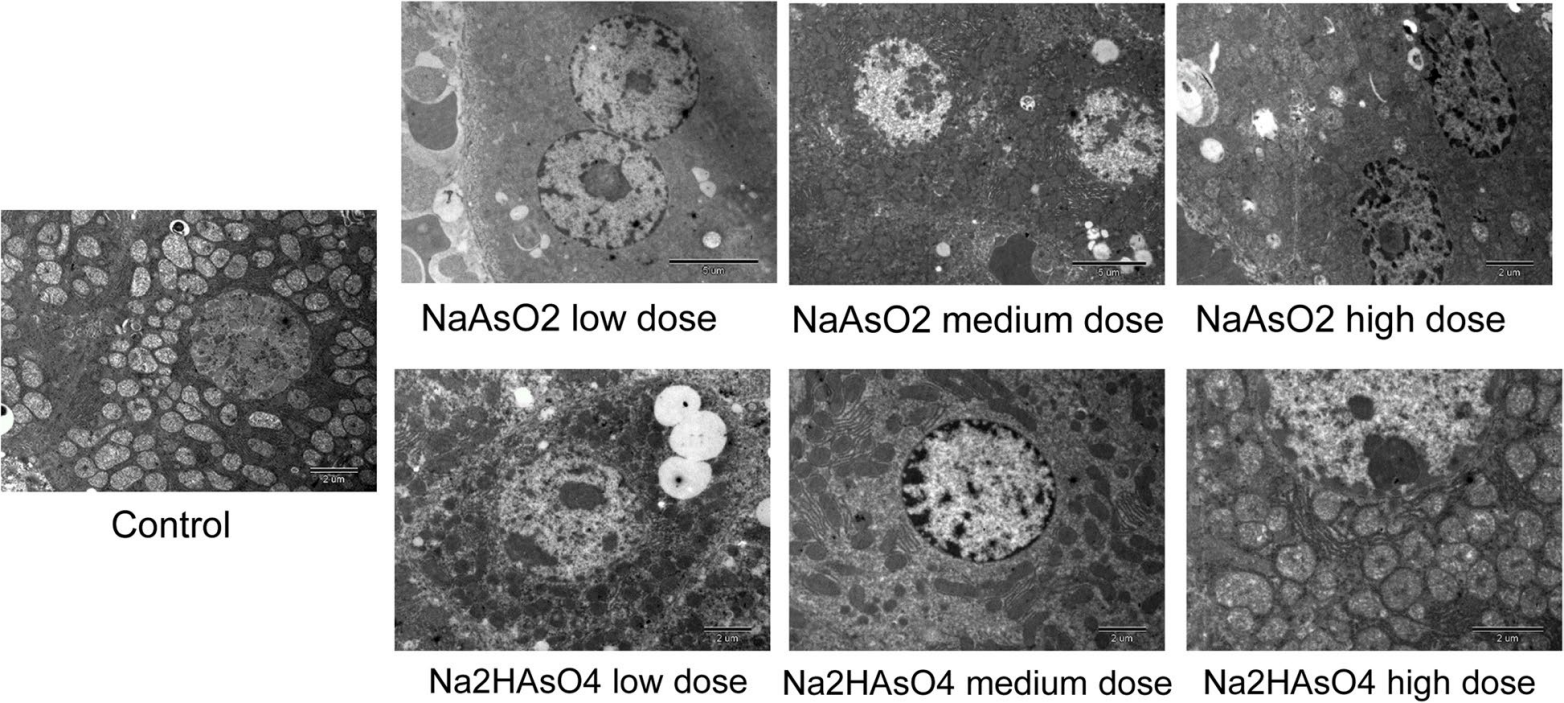
Pathological changes of live tissue. Electron microscopy was performed to assess the pathological changes of liver tissue from each group. Scale bar = 2 μm or 5 μm as indicated

Electron microscopy observations showed that in the liver section of the control group (Fig. 1), the hepatocytes had round nuclei, more euchromatin, less heterochromatin, a few fat droplets, abundant endoplasmic reticulum, and normal bile ducts. However, in the iAs^3+^ and iAs^5+^ high-dose groups, the core of hepatocytes was irregular; the heterochromatin increased and appeared to be granular; lipid droplets increased; a few mitochondria had vacuolar ends, and nucleoli were edged; and, collagen was seen in the intercellular space. There were fibrous hyperplasia, dense membrane-like material in the bile duct area, widening of the hepatocyte space, general swelling of cells, reduced matrix density, loose cristae arrangement, obvious increase of similar substances in liver cells, and, collagen hyperplasia in the Disse space. In the iAs^3+^ medium/low-dose groups and the iAs^5+^ medium/low-dose groups, round nuclei, more euchromatin, less heterochromatin, and slight lipid droplets were observed in the cells, and the cells were basically normal, with a few swollen cells. There was no obvious difference in electron microscope observation between iAs^3+^ and iAs^5+^ groups at the same dose.

### Comparison of arsenic DMA and MMA in rat liver

**Fig. 2:**
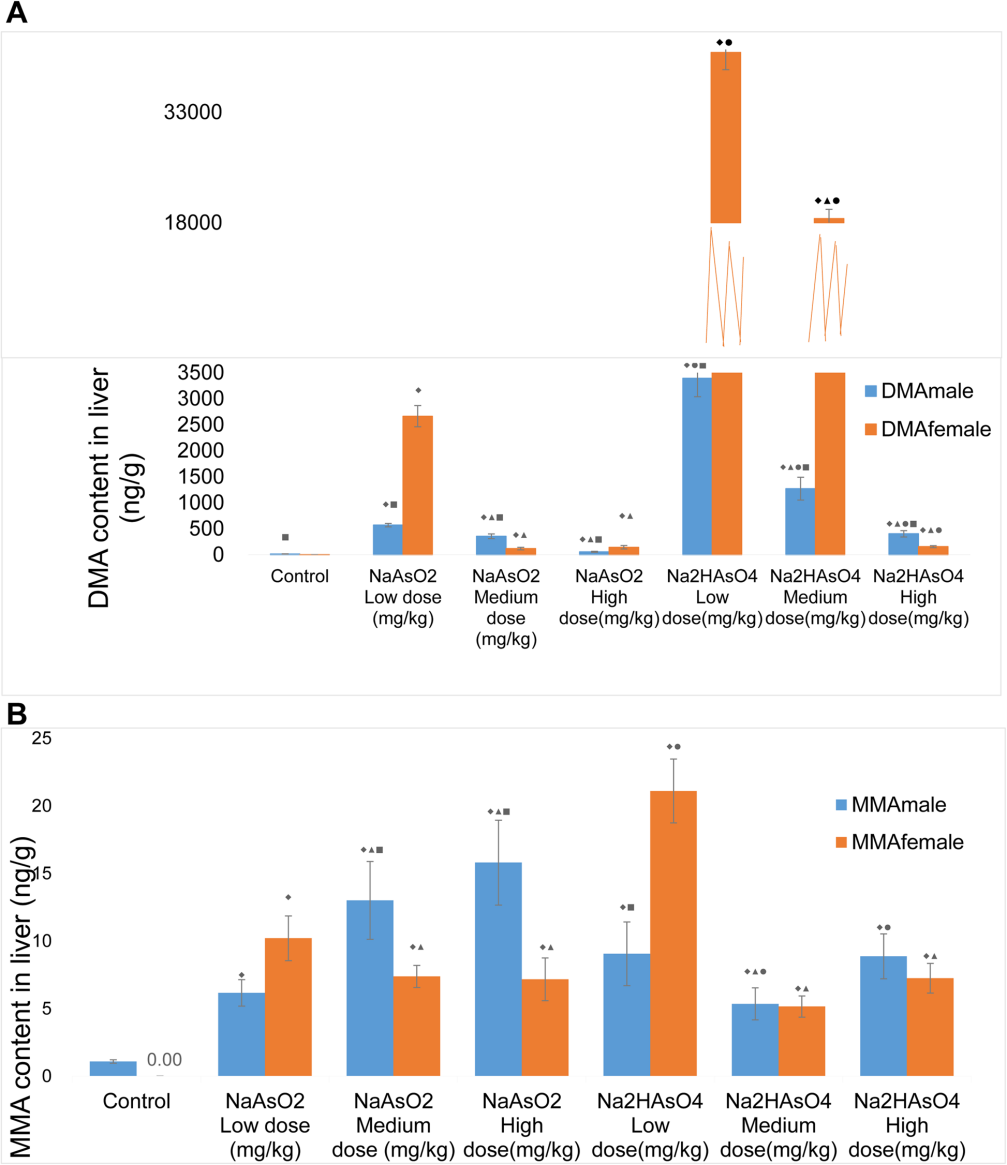
Levels of MMA and DMA in liver tissues after arsenic exposure in each dose group. **A**: MMA content. **B**: DMA content. Data were expressed as mean ± standard deviation and analyzed by one-way variance analysis followed by LSD-t test or SNK method. ◆Compared with normal control group, *P* < 0.05; ▲Compared with low-dose group, *P*< 0.05; ●Compared with different arsenic compounds in the same dose group, *P* < 0.05; ■Comapared the male and female animals in the same group *P* < 0.05

The ASE method was used to extract the arsenic metabolites in the liver, and the arsenic metabolites, including MMA and DMA, in the liver were analyzed by high performance liquid chromatography-hydride generation atomic fluorescence spectrometry. The results showed that in each group, DMA and MMA in the liver of male and female rats were significantly higher than those in the normal control group (Fig. 2A and B, *P* < 0.05). Compared with the low-dose group, except for the male iAs^5+^ high-dose group, the differences in MMA and DMA in other groups were all statistically significant (*P* < 0.05). Except that the MMA in the high- and medium-dose male groups was up-regulated compared to the low-dose group, the DMA and MMA of the other high- and medium-dose groups decreased with the increase in the arsenic exposure dose compared with the low-dose groups. When comparing the effects of different arsenic compounds in the same dose group, the MMA differences between the female iAs^5 +^ low-dose group and the male iAs^5 +^ high-dose and medium-dose groups were statistically significant. The DMA levels were all statistically significant between male and female rats (*P* < 0.05). Compared with male and female animals in the same group, the MMA in groups of iAs^3+^ high, medium, and iAs^5+^ low was all statistically significant, and the differences in DMA were statistically significant (*P* < 0.05). However, the MMA of male rats was higher in iAs^3+^ high and medium dose groups than that of female rats, and the DMA of male rats was lower than that of female rats. Thus, females may have high methylation ability and more synthetic DMA.

### Effect of gender on the relative expression of As3MT mRNA in rats

**Fig. 3:**
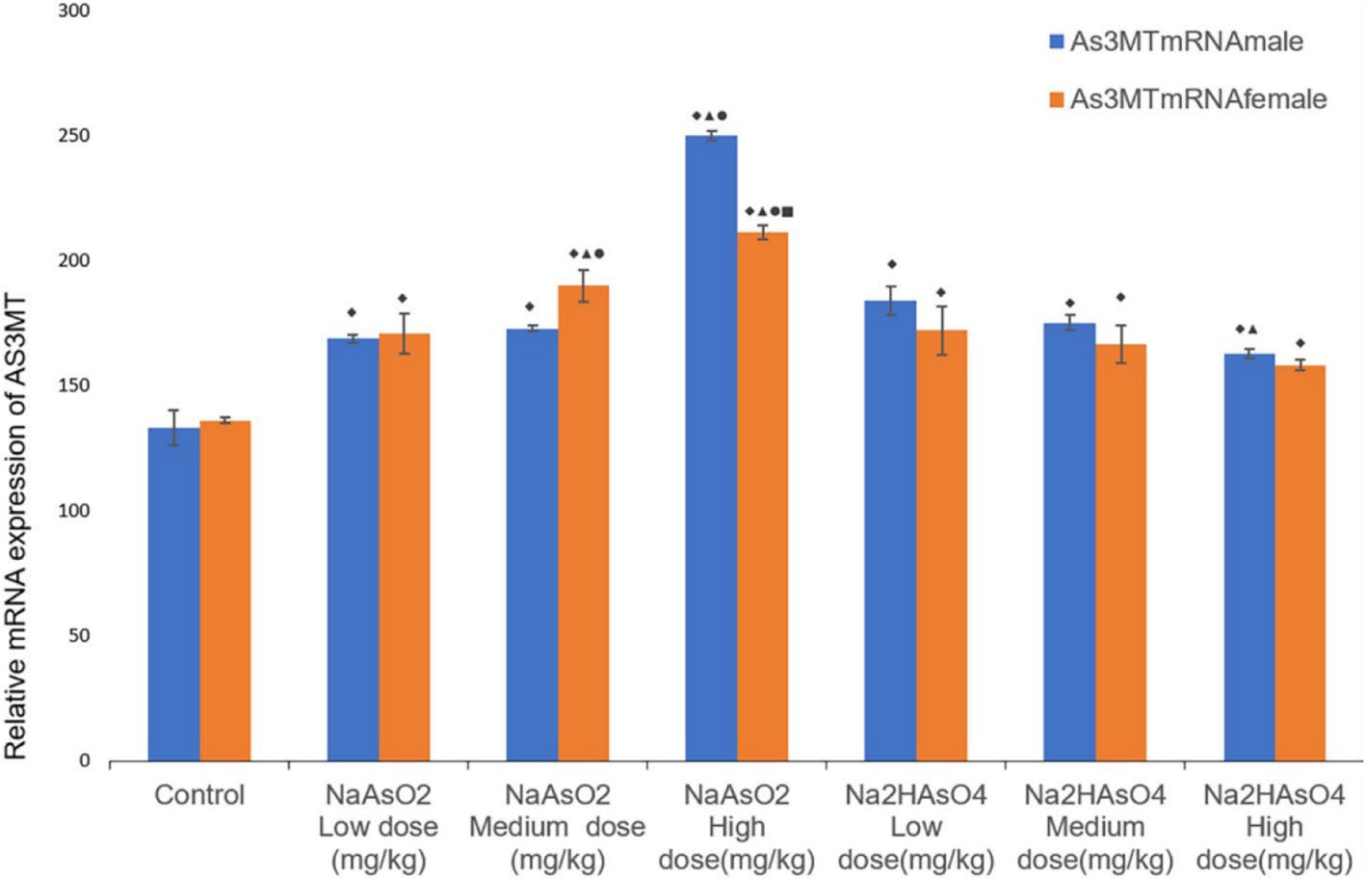
Relative mRNA expression of AS3MT in rats exposed to arsenic in each group. RT-qPCR was used to detect mRNA level. Data were expressed as mean ± standard deviation and analyzed by one-way variance analysis followed by LSD-t test or SNK method.◆Compared with normal control group, *P* < 0.05; ▲Compared with low-dose group, *P* < 0.05; ●Compared with different arsenic compounds in the same dose group, *P* < 0.05; ■Compared the male and female animals in the same group *P* < 0.05

RT-qPCR was used to detect the expression of *As3MT* mRNA in the liver. After different doses of iAs^3+^ or iAs^5+^ treatment, As3MT in both male and female rats in each group was higher than that in the normal control group (*p* < 0.05) (Fig. 3). Compared with the low-dose group, only the same test substance in the female iAs^3+^ high dose group, iAs^3+^ medium dose group, male iAs^3+^ high dose group, and iAS^5+^ high dose group had statistical differences (*P* < 0.05). Comparing different test substances in the same dose group, only the female iAs^3+^ high dose group, the female iAs^3+^ medium dose group, and the male iAs^3+^ high dose group had statistical differences (*P* < 0.05). Compared with male and female animals in the same group, the expression of *As3MT* mRNA in iAs^3+^ high dose group was higher in males than females (*P* < 0.05).

### Effects of gender on MPO activity in the liver of rats

**Fig. 4:**
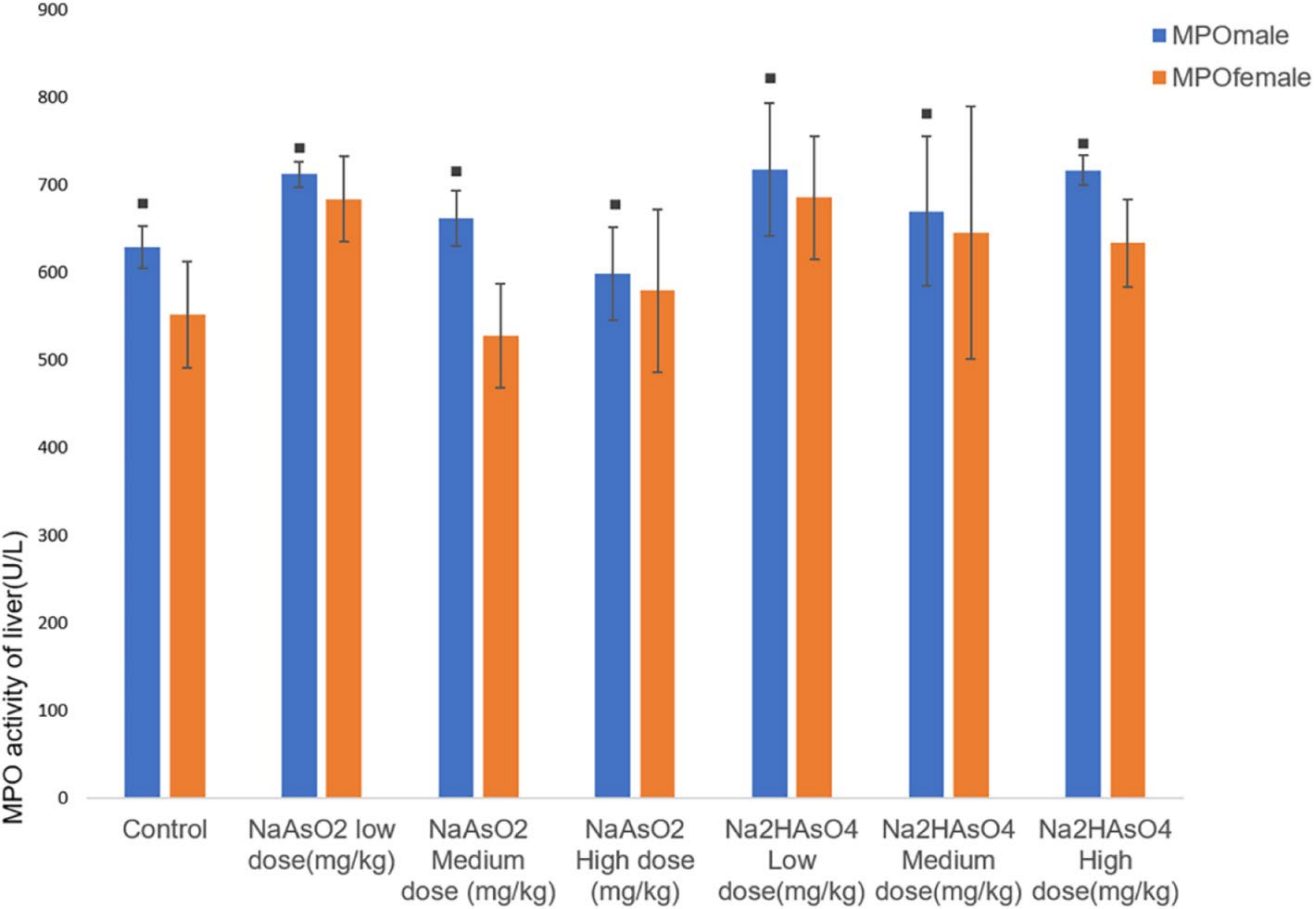
Liver MPO activity in each group. Data were expressed as mean ± standard deviation and analyzed by one-way variance analysis and LSD-t test. ◆Compared with normal control group, *P* < 0.05; ▲Compared with low dose group,*P* < 0.05; ●Compared with different  arsenic compounds in the same dose group, P < 0.05; ■Compared the male and female animals in the same group, *P* < 0.05

The MPO activity in the liver was determined by ELISA. Compared with the control group, the MPO activity of the iAs^3+^ low-dose group and the iAs^5+^ high-dose group increased (*P* < 0.05). Besides, the difference between male and female in the same group was statistically significant (*P* < 0.05, Fig. 4).

### Effects of gender on SAM in the liver of rats

**Fig. 5:**
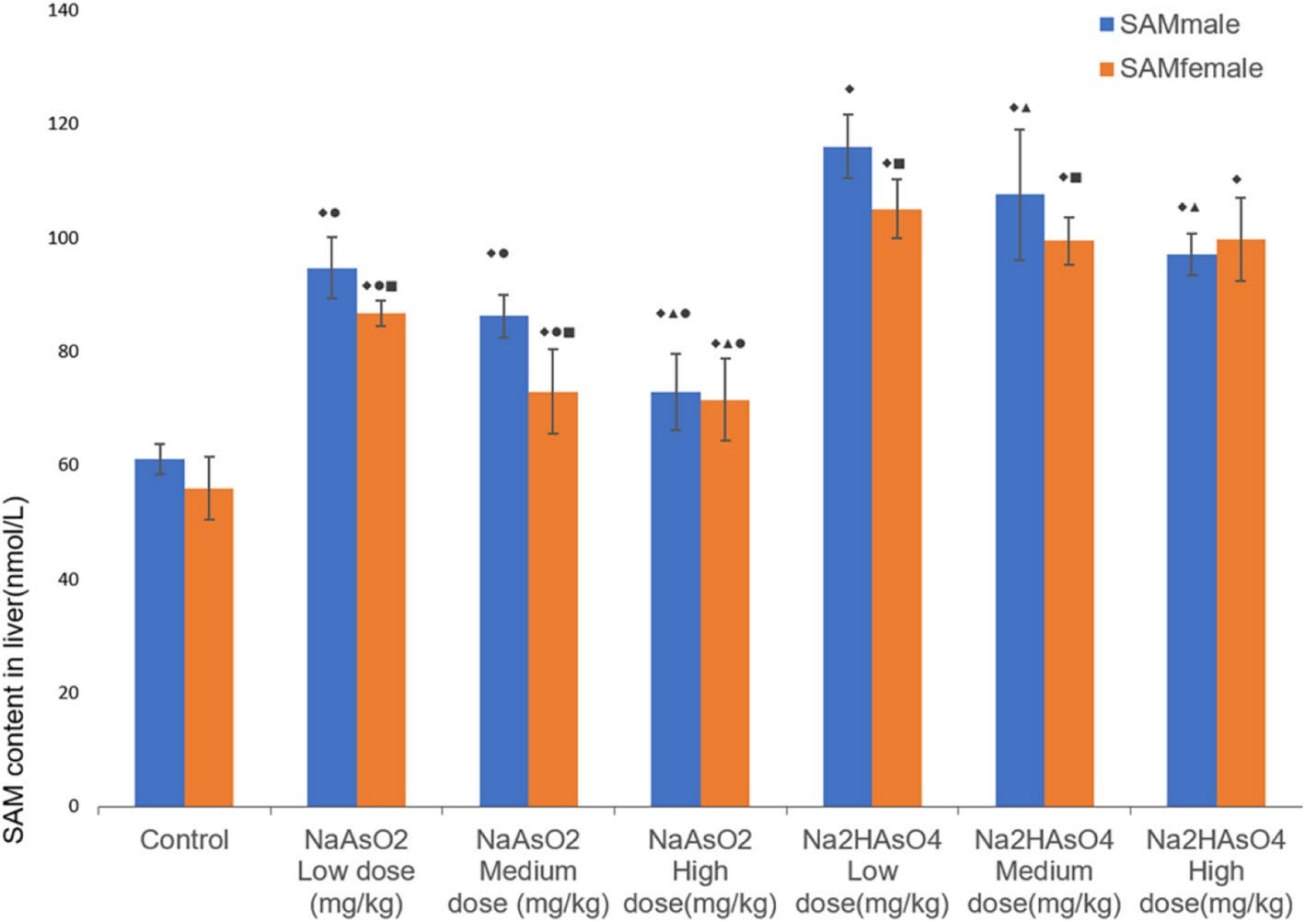
The content of SAM in the liver in each group. Data were expressed as mean ± standard deviation and analyzed by one-way variance analysis and LSD-t test. ◆Compared with normal control group, *P* < 0.05; ▲Compared with low-dose group, *P* < 0.05; ●Compared with different arsenic compounds in the same dose group, *P* < 0.05; ■Compared the male and female animals in the same group, *P* < 0.05

The SAM level in the liver was also determined by ELISA. Compared with the control group, the SAM activity of each iAs^3+^ or iAs^5+^ group was significantly increased (*P* < 0.05) (Fig. 5). Compared with the iAs^3+^ low-dose group, the activity of SAM in the iAs^3+^ high-dose group was lower (*P* < 0.05). The activity of SAM was lower in iAs^5+^ high and medium dose groups than that in iAs^5+^ low dose groups (*P* < 0.05). In the iAs^3+^ medium/low-dose groups, and iAs^5+^ medium/low-dose groups, the SAM activity of females was lower than that of males (*P*< 0.05, Fig. 5).

### Effects of gender on ARR activity in the liver of rats

**Fig. 6:**
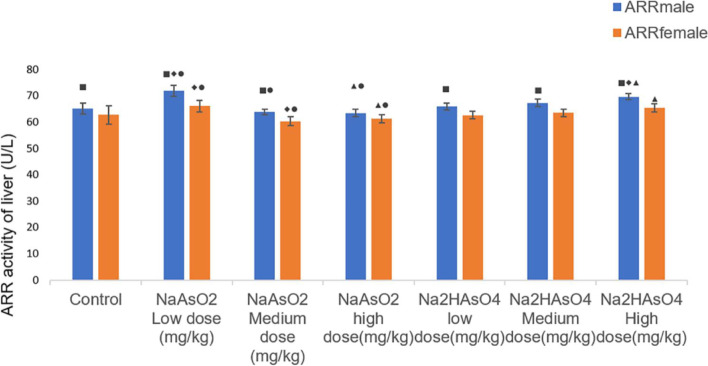
Liver ARR activity in each group. Data were expressed as mean ± standard deviation and analyzed by one-way variance analysis and LSD-t test. ◆Compared with normal control group, *P* < 0.05; ▲Compared with low-dose group, *P* < 0.05; ●Compared with different arsenic compounds in the same dose group, *P* < 0.05; ■Compared the male and female animals in the same group *P* < 0.05

As shown in Fig. 6, the ARR activity of the iAs^3+^ low-dose group was higher than that of the normal control group, while the ARR activity of the male iAs^5+^ high-dose group was higher than that of the normal control group. Additionally, the ARR activity of the iAs^3+^ high-dose group was lower than that of the iAs^3+^ low-dose group. The ARR activity of iAs^5+^ high-dose group was higher than iAs^5+^ low-dose group. In the same group, compared between male and female, except for the iAs^3+^ high-dose group, the ARR activity of males in other groups was higher than that of females (Fig. 6).

### Effect of gender on PNP level in the liver of rats

**Fig. 7:**
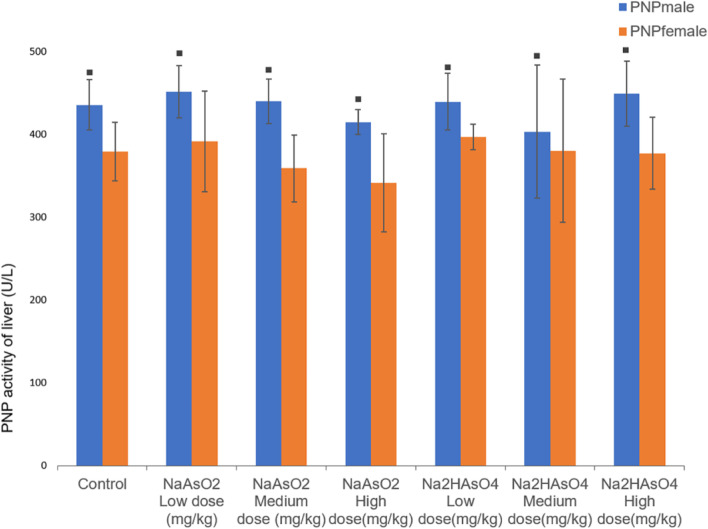
PNP activity in the liver in each group. Data were expressed as mean ± standard deviation and analyzed by one-way variance analysis and LSD-t test.◆Compared with normal control group, *P* < 0.05; ▲Compared with low-dose group, *P* < 0.05; ●Compared with different arsenic compounds in the same dose group, *P* < 0.05; ■Compared the male and female animals in the same group *P* < 0.05

The analysis of PNP activity in the liver of each group of rats found that in the same group of animals, the PNP activity of male rats was higher than that of female rats (*P* < 0.05, Fig. 7).

### Effects of gender on the PK activity in the liver of rats

**Fig. 8:**
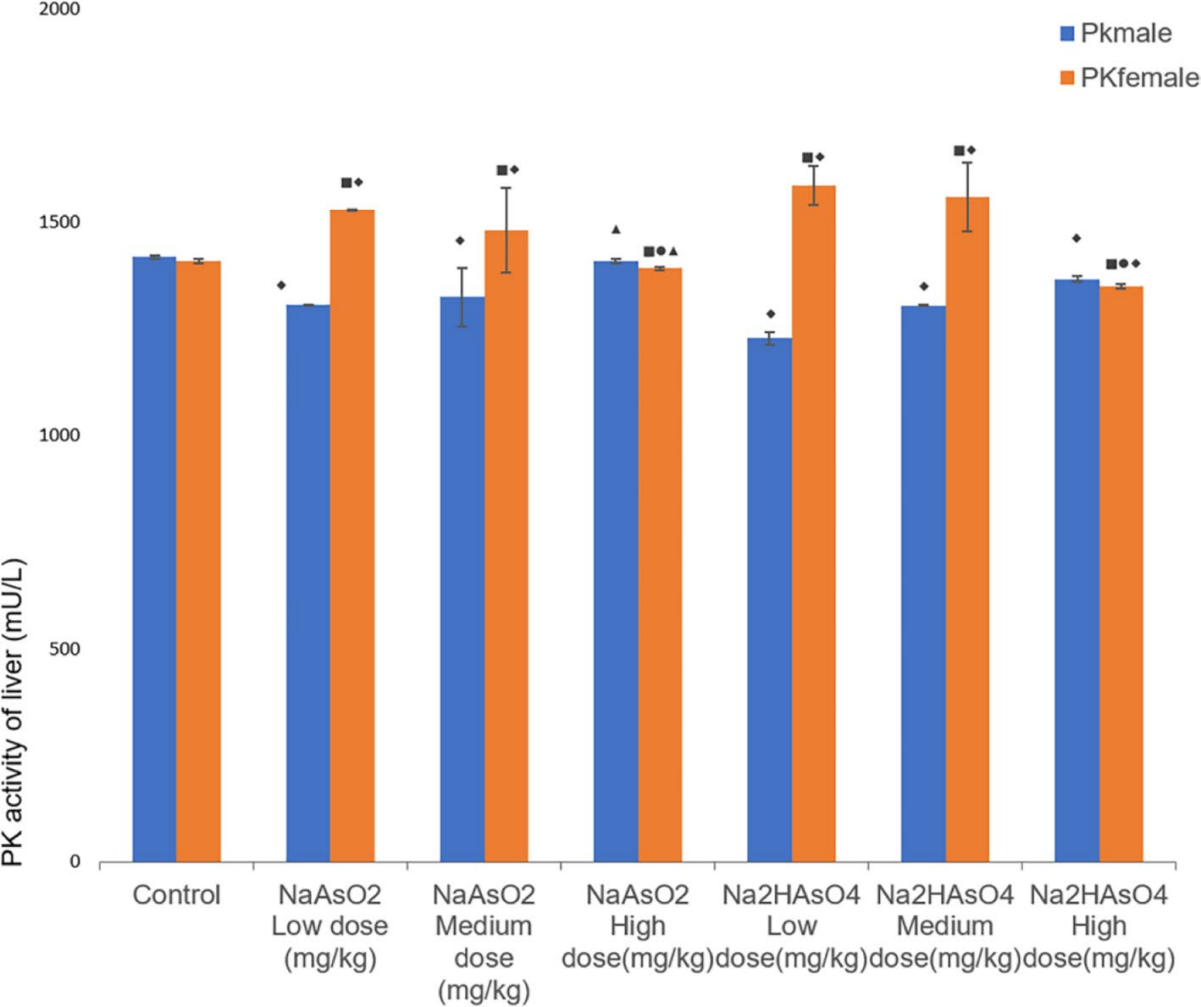
Liver PK activity in each group. Data were expressed as mean ± standard deviation and analyzed by one-way variance analysis and LSD-t test. ◆Compared with normal control group, *P* < 0.05; ▲Compared with low-dose group, *P* < 0.05; ●Compared with different arsenic compounds in the same dose group, *P* < 0.05; ■Compared the male and female animals in the same group *P* < 0.05

The PK activity in the liver was determined by ELISA. After exposure to different doses of iAs^3+^ or iAs^5+^, except for the iAs^3+^ high-dose group, the PK activity in remaining groups was statistically different from that in the control group (*P* < 0.05) (Fig. 8). Compared with the low-dose group, the iAs^3+^ high-dose group had statistical difference (*P* < 0.05). In addition, there was a statistical difference between the female iAs^3+^ high-dose group and the iAs^5+^ high-dose group (*P* < 0.05). In the same group, comparison between male and female, except for the control group, PK activity in other groups of female rats was higher than that of male rats (*P* < 0.05, Fig. 8).

### Effect of gender on NAD level in the liver of rats

**Fig. 9:**
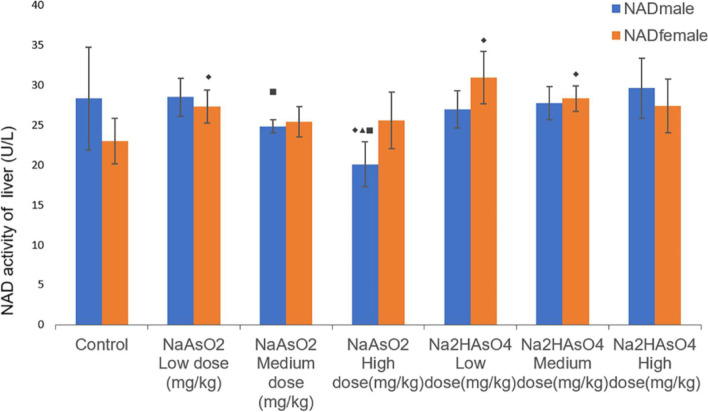
Liver NAD activity in each group. Data were expressed as mean ± standard deviation and analyzed by one-way variance analysis and LSD-t test.◆Compared with normal control group, *P* < 0.05; ▲Compared with low-dose group, *P* < 0.05; ●Compared with different arsenic compounds in the same dose group, *P* < 0.05; ■Compared the male and female animals in the same group *P* < 0.05

ELISA was performed to determine the NAD level in the liver. Compared with the control group, there was a statistical difference in NAD of female iAS^3+^ low dose group, the iAS^5+^ low/medium dose group and the male iAS^3+^ low dose group (*P* < 0.05) (Fig. 9). Compared with the low-dose group, the activity of NAD in the iAS^3+^ high-dose group decreased (*P* < 0.05). In comparison between male and female, the NAD activity of females in iAs^3+^ high and medium dose groups was higher than that of males (*P* < 0.05). These results indicate that under the same iAs^3+^ exposure, arsenic inhibited NAD activity in males, and promoted NAD activity in females.

## Discussion

The results of this study showed that after exposure to iAs^3+^ or iAs^5+^, the rat liver of high dose groups had irregular cell cores, increased heterochromatin and granular nucleoli, dense membrane-like material in the bile duct area, swelling of individual cells, and reduced matrix density. This pathological changes of the liver are consistent with previous reports [16–18]. The content of DMA and MMA in the liver of male rats and female rats in each group was significantly higher than that in the normal control group, reflecting that arsenic exposure may affect DMA and MMA content. DMA and women are positively correlated, and there are gender differences in arsenic metabolism [19]. Herein, the DMA content of male rats was lower than female rats in the same group. This is consistent with the conclusion of a study that women had higher urine excretion levels of DMA than men [20], the possible mechanism may be that the solubility of MMA in urine is lower than that of DMA, which means that MMA is less excreted in urine and accumulates in target organ. It has been shown that MMAs^3+^ is the most toxic As^5+^ and As^3+^ metabolite in mammals [21]. Studies have shown MMA is more toxic than DMA [22, 23]. The MMA content of male rats was greater than that of female rats in the same group. This may be the metabolic basis for the gender difference in arsenic poisoning. Men may be more sensitive to arsenic damage than women [24]. The above results also showed that arsenic in males was more pathogenic than that in females. It is inferred that arsenic metabolites may have higher toxic effects on liver tissues of male rats than on those of females. It has been reported that women excrete higher amounts and percentages of DMA with lower iAs and MMA than men, suggesting that women possess an overall increased capacity to methylate As [12, 25–27]. This conclusion is also consistent with the results of several previous studies [28, 29].

The liver is the main site of arsenic methylation metabolism. Study has found that in boys, there was a positive correlation between the AS3MT-Met287Thr polymorphism and %MMA; while there was a negative correlation between AS3MT-Met287Thr and the second methylation profile [30]. In this study, we explored the effects of different valences of arsenic on the expression of As3MT in rat liver. The results showed that the expression of As3MT in the high, medium, and low dose groups of iAs^3+^ and iAs^5+^ were higher than those in the control group, indicating that arsenic exposure can increase expression of As3MT. The expression level of As3MT in the high and medium dose groups of iAs^3+^ was higher than that in the low dose group, and the expression level gradually increased as the dose of iAs^3+^ increased. The expression level of As3MT in the iAs^5+^ high-dose group was lower than that in the low-dose group, and the expression level gradually decreased as the iAs^5+^ dose increased. It shows that there may be a positive correlation and a negative correlation between the exposure of iAs^3+^ and iAs^5+^ and the expression of As3MT, and there may be a certain dose–effect relationship, which was contrary to the study using frogs [31]. The expression of As3MT in the liver in the iAs^3+^ high dose group was higher than that of the iAs^5+^ high-dose group. Thus, it can be inferred that arsenic with different valences has different arsenic methylation patterns in the body. The arsenic methylation and level of arsenic would accelerate the excretion of methylated arsenic through urine [32, 33], but it can also enhance the potential genotoxicity and long-term effects [34], which is consistent with the same metabolic pattern of low-dose iAs^5+^ in liver. Obviously, the high dose of iAs^5+^ in the liver inhibited the expression of As3MT to a certain extent. Compared with male and female animals in the same group, the relative expression of As3MT mRNA in male rats in the iAs^3+^ high dose group was higher than that in female rats, indicating that arsenic has different arsenic methylation patterns in different sexes. This result reflects the tension or disorder of arsenic methylation and detoxification pathways in males, which is consistent with previous study [35].

In this study, it was found that compared with the control group, both the iAs^3+^ low-dose group and the iAs^5+^ high-dose group promoted the increase of MPO activity in rats. MPO is present in the cell and all three subtypes of MPO can form a strong complex with DNA to prevent damage during oxidation and ensure the normal differentiation of cell functions [36]. The increase in MPO content caused by arsenic poisoning may also contribute to lipid peroxidation and oxidative cell damage [37]. In this study, MPO activity of male rats was greater than that of female rats, which is similar to the results of another study [38]. These results indicate that male rats and female rats may have different lipid peroxidation processes. It has been found that after external environmental stimuli, the activity of MPO decreases and the production of reactive oxygen species is also reduced, thereby reducing the damage of arsenic to the body [39]. Therefore, the higher MPO activity in males will increase the damage of arsenic to the body.

The results showed that SAM in the liver of rats after exposure to iAs^3+^ or iAs^5+^ was significantly higher than that in the control group, indicating that different valences of arsenic affected the activity and level of SAM to change the methylation of DNA and histones [40]. Comparing with the low-dose group, the content of SAM in iAs^3+^ high-dose group, iAs^5+^ high- and medium dose groups was lower, and the content gradually decreased with increasing dose. It can be inferred that there is a certain dose–effect relationship between the exposure of iAs^3+^ and iAs^5+^ and the content of SAM in the body, that is, the higher the arsenic exposure, the lower the SAM level [41, 42]. Additionally, we observed that compared with different valences, iAs^3+^ had higher SAM excessive consumption or failure than iAs^5+^. Therefore, the arsenic methylation of iAs^5+^ is relatively sufficient, which will generate more MMAs and DMAs [43]. During this process, there will be more active free radicals, resulting in abnormal DNA methylation, leading to stronger genotoxicity and exerting long-term effects such as carcinogenesis [41, 44]. Compared with male and female animals in the same group, the SAM activity of female rats in the iAs^3+^ medium and low-dose groups and iAs^5+^ medium and low-dose groups was lower than that of male rats. This indicates that the same arsenic exposure may exert greater acute toxicity, stronger genotoxicity and long-term effects such as carcinogenesis in males.

Tseng et al. reported that in adults, women had a better arsenic methylation capacity than men, and this difference had been partially explained by the stimulating effect of estrogens on the synthesis of choline, which is involved in the remethylation of homocysteine to methionine, a precursor of S-adenosylmethionine, the methyl donor for arsenic methylation [12, 45]. ARR is the key enzyme that regulates these two detoxification processes, and it is the rate-limiting enzyme in the process of arsenic methylation. It plays an important role in the entire metabolic process and helps reveal the process of arsenic methylation. Exploring the mechanism of arsenic toxicity is of vital importance. We also found that the activity of ARR in the iAs^5+^ high-dose group and iAs^3+^ low-dose group was higher than that in the control group. The ARR activity of the iAs^3+^ high-dose group was lower than that of the iAs^3+^ low-dose group, and the ARR activity of the iAs^5+^ high-dose group was higher than that of the iAs^5+^ low-dose group. Therefore, exposure to high doses of arsenic can lead to changes in ARR activity, and the effect of iAs^5+^ on ARR shows a certain dose relationship. On the other hand, the ARR activity of the iAs^5+^ high dose/medium dose group was higher than that of the iAs^3+^ high dose/medium dose group; and that of the iAs^5+^ low dose group was lower than that of the iAs^3+^ high dose group. This may be related to the different mechanisms by which arsenic of different valences inhibit ARR activity. ArrAB complex is a bacterial heterodimer or surface-anchored ararsenic reductase whose methylation is also thought to be the mechanism of arsenic resistance in bacteria, fungi and mammals [46, 47]. Comparison between male and female animals in the same group, except for the iAs^3+^ high dose group, the ARR activity of males in the other groups was higher than that of females, indicating that the males may compensatively stimulate the body to produce more ARR.

As for PNP, we found that male rats could promote the compensatory increase of PNP activity more than female rats. PNP is an important pathway for the reduction of arsenate to arsenite in mammalian systems [48]. The increased expression of PNP mRNA will increase the level of MMA in the urine of the population, thus exerting greater long-term effects such as genotoxicity or carcinogenesis and mutagenesis. At least one G allele of PNP RS3790064 increases the risk of arsenic-related skin lesions in persons exposed to arsenic, and variations in PNP predisposes individuals exposed to high doses of inorganic arsenic to arsenic-induced skin lesions [49].

PK plays an important role in cell metabolism [50]. The results of this study showed that the activity of PK in the control group was higher than that in the iAs^5+^ high-dose group, indicating that high-dose arsenic exposure can cause abnormal PK activity. The PK activity of the iAs^3+^ group was higher than that of the iAs^5+^ group. It can be inferred that arsenic with different valences may affect PK activity through interfering with the glycolysis process, leading to abnormal cell energy metabolism [10]. Compared with males and females of the same group of animals, the PK activity of females was higher than that of males, suggesting that arsenic may change the glycolysis process by inhibiting the activity of PK in males, which may then affects the body's energy supply and causes greater toxicity.

Furthermore, we found that after exposing to different doses of iAs^3+^ or iAs^5+^, the NAD of male iAs^3+^ high-dose group was lower than that of control group. It may be caused by the interference of physiological and biochemical process by high-dose iAs^3+^, which leads to insufficient cellular ATP synthesis required for metabolism [51, 52]. There was a statistically significant difference between the different doses of iAs^3+^, suggesting that lower dose of iAs^3+^ promoted the activity of NAD, it may be due to the involvement of NADH in the oxidation of iAs^3+^ to iAs^5+^ [53]. Study has shown that arsenic trioxide inhibits nicotinamide phosphoribosyl transferase, thereby depleting NAD [54]. In addition, the difference between males and females in each group was statistically significant, indicating that arsenic promoted the NAD activity of females and inhibited the NAD activity of males. Moreover, arsenic also inhibited glycolysis and caused abnormal metabolism, thus exerting a toxic effect [51, 52].

This study is limited in that the underlying mechanism was not analyzed. Further studies are needed.

## Conclusion

In conclusion, most studies only observed and analyzed the relationship between a single gene and arsenic. This study analyzed the content and activity of multiple arsenic metabolism-related enzymes in the liver of rats of different genders. It is found that arsenic had more toxic effects on male animals than females. The effect of estrogen may be the reason why the biotransformation rate of arsenic in female rats is higher than that in male rats. In the future, it is necessary to further study the combination of multiple arsenic metabolism-related genes and the interaction between genes and the environment.

## Data Availability

The data used and/or analysed during the current study are available from the corresponding author on reasonable request.
